# Lithotrity

**Published:** 1831

**Authors:** 


9. *Lithotrity.*—Dr. Civiale communicated to the Academy of Science, (Jan. 24, 1831,) a report of the cases of lithotrity, recently placed under his notice. He attributes the failure of the process, in most cases where failure has occurred, to the imperfection of the instruments employed. He has operated successfully on 152 cases, and has seen other practitioners equally successful in 13 cases.—*Ib. May* 1831.

